# Mercy Hospital—Opthalmological Ward

**Published:** 1871-10

**Authors:** 


### MERCY HOSPITAL.—OPTHALMOLOGICAL WARDS.

---

Service of PROF. JONES.

---

CASE I. PTOSIS.—In the falling of the eyelid which constitutes ptosis, the patient can often raise the lid by an effort, as in the present case; but the exertion exhausts the nervous energy, and the act cannot be repeated for a time. Ptosis has various causes, but in the present instance it originates from a condition bordering on paralysis. The man had a severe fall four years ago, and his occupation has required him to do heavy lifting. He is seen to be in an anæmic condition.

If other means fail, the usual operation of removing an elliptical piece of the lid must be resorted to, but milder methods will be tried. In many cases tonic treatment proves very efficient. Strychnia and iron are directed for this patient, with external irritation to excite the circulation in the part. This course has given such satisfactory results as to justify the expectation of relief to the patient without operation.

---

CASE II. STAPHYLOMA.—This gentleman exhibits an instance of staphyloma resulting from an injury, the kick of a horse. He was operated on last spring, and the cornea amputated in such a way as to leave a good stump for an artificial eye. He left the Hospital in good condition, but the thinning of the cornea has again allowed the contents to bulge forward, and he has returned to have another portion removed. Formerly the eye was removed in such cases, but by preserving a stump with the muscles attached, movements of the artificial eye are secured. For the operation the patient was put under the influence of chloroform, and a speculum introduced between the eyelids to keep them apart. Large curved needles were introduced through the cornea, behind the cornea, in order to prevent the escape of the vitreous humor; the cornea was then cut out, and the stump cut down to the size best adapted to the artificial eye which is to be worn; then, with smaller needles, the incision was sewed up smoothly, and the patient directed to be kept quiet.

---

CASE III. CONJUNCTIVITIS WITH GRANULATED LIDS.—This man's eyes began to look red and feel inflamed about a year ago; now there is conjunctivitis, with granulated lids. No ulceration of the cornea, which is so frequent and serious a result, has yet occurred; the upper lid is worse than the lower, according to the usual custom. In an earlier stage a weak solution of sulphate of zinc, or of borax, sometimes with morphia, would have been used as a wash. But since the acute stage is passed the granulated lids are to be touched with a piece of sulphate of copper which has been shaved smooth and then polished on chamois-leather. The lids are easily everted for this operation by taking hold of the eyelashes, at the same time telling the patient to look down towards the floor, then putting a pencil on the lid and lifting it up by the lashes.

